# Food self-sufficiency: Managing the newly-opened tidal paddy fields for rice farming in Indonesia (A case study in West Kalimantan, Indonesia)

**DOI:** 10.1016/j.heliyon.2023.e13839

**Published:** 2023-02-20

**Authors:** Muhammad Hatta, Sulakhudin, Rusli Burhansyah, Gontom C. Kifli, Dina O. Dewi, Juliana C. Kilmanun, Dadan Permana, Khojin Supriadi, Riki Warman, Hozin Azis, Putri Tria Santari, Dwi P. Widiastuti

**Affiliations:** a National Research and Innovation Agency, B.J. Habibie Building 15th-24th Floor, Jl. M.H. Thamrin No. 8, Jakarta Pusat 10340, Indonesia; b Department of Soil Science, Faculty of Agriculture, Tanjungpura University, Jl.Prof. Hadari Nawawi, Pontianak, West Kalimantan 78121, Indonesia

**Keywords:** Newly-opened rice field, Farmers' collaboration, Farmer farming facilitation, Rice productivity, Farmers' income, Food self-sufficiency

## Abstract

The Indonesian government continues to develop a sustainable food self-sufficiency program by increasing national food security through an extension program. One of the instruments is by opening new rice fields. The area of new rice fields in Indonesia is 222,442 ha spreading on the islands of Sumatra, Kalimantan, and Papua. This new rice field is estimated to produce 1.2 million tons of rice per year. In the case of West Kalimantan Province, it has opened new rice fields cover an area of 23,384 ha, mostly in tidal lands. Expansion of newly-opened rice fields does not increase land productivity. Moreover, rice productivity in the newly-opened paddy fields is only an average of 2 t ha^−1^. The low rice productivity is caused by biophysical factors of land in agriculture, and social-economic, and institutional factors of farmers at the village level. Therefore, it is necessary to have a rice farming model in newly-opened rice fields involving farmer groups, researchers, agricultural extension agents, government agencies, the private sector, and banks. The purpose of this study was to present a sustainable rice farming model in the newly-opened tidal rice fields. The results of this study showed that application of the rice farming model in newly-opened tidal rice fields could increase rice productivity from 2 to 5.7 t ha^−1^ and farmer income of IDR 10.6 million, involving good collaboration among farmer groups and farmer economic organizations supported by banks for sustainability.

## 1 Introduction

The prolonged COVID-19 pandemic has led to looming food insecurity and food crisis in several countries [1]. Food and Agriculture Organization (FAO) representatives in Indonesia report that during the COVID-19 pandemic which has led to an economic recession, it is estimated that at least 132 million people will suffer from hunger which will add to new concerns in the food and agriculture sector in the coming few years. This condition is urgently needed to be tackled amicably in time, especially for Indonesia regarding national food availability. FAO is very concerned about the potential impact of COVID-19 related to food security and the global food crisis. FAO as a world food institution is obliged to maintain and anticipate the impact of the global COVID-19 pandemic through 2 aspects, namely (1) strengthening food security and (2) overcoming the humanitarian crisis. COVID-19 vaccination activities can encourage farmers to start farming businesses and maintain the food distribution chain [2].

**Fig. 1:**
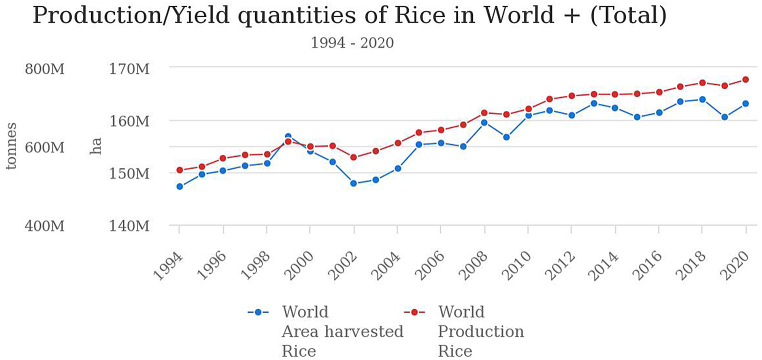
Production/yield of rice, paddy in the world [3].

In the last ten years, the area and food production in the world, especially rice, has increased. The planted area and world rice production in 2018 have reached nearly 170 million hectares with production approaching 800 million tons [3]. However, starting in 2019, the world's planting area and rice production have decreased (Fig. 1) [3].

Agricultural development in Indonesia will face heavy challenges, especially in meeting national food needs such as rice, which each year will continue to increase in line with the increasing population and decreasing paddy fields. The total population of Indonesia in 2020 is approximately 268.1 million people with a growth rate of 1.31% per year [4,5]. Meanwhile, the area of paddy fields in Indonesia has decreased from 7.7 million hectares in 2013 to 7.1 million hectares in 2018; and the area of paddy fields has been revised to 7,463,948 ha in 2019 [5].

According to these data, Indonesia's rice fields have decreased by an average of 39,342 ha per year. Presumably, paddy fields have been converted from agriculture to non-agricultural utilization. This condition needs to be observed and aspired to meet national food needs, so there will be no catastrophic food shortages in the coming years. One way to fulfill food demands is by utilizing marginal lands such as tidal swamps for food crop farming.

Currently, the area of swamps in Indonesia is 32.64 million hectares, of which 9.89 million hectares are tidal swamps in mineral soils that have the potential for the development of rice crops [6] (Table 1). Tidal swamplands in Indonesia are mostly scattered on the islands of Sumatra, Kalimantan, and Papua. The development of rice farming in tidal swamplands has long been carried out in Indonesia through intensification and extensification programs with yields around 2–4 t ha^−1^ [6]. Extensification programs have been carried out by opening new rice fields, among others, on the island of Kalimantan. Kalimantan has around 3 million hectares of tidal lands; and the largest area is in West Kalimantan, i.e. 932,839 ha [6] (Table 2).

**Table 1:** Distribution of swamplands in Indonesia.

Island	Tidal swampland (ha)		Lowland swamp (ha)		Total (ha)
	Mineral soil	Peat soil	Mineral soil	Peat soil	
Sumatera	3.526.546	962.911	1.989.734	4.887.650	11.366.841
Java	195.375	–	60.403	–	255.778
Kalimantan	3.045.938	467.813	2.671.843	4.075.549	10.261.143
Sulawesi	370.563	1.044	153.059	23.739	548.405
Maluku	143.183	–	24.582	–	167.765
Papua	2.605.128	360.486	4.426.373	2.651.325	10.043.313
Total (ha)	9.886.734	1.792.255	9.325.995	11.638.263	32.643.246

**Table 2:** Distribution of swampland on Kalimantan island.

Provinsi	Tidal swampland (ha)		Lowland swamp (ha)		Total (ha)
	Mineral soil	Peat soil	Mineral soil	Peat soil	
West Kalimantan	932,839	360,117	485,830	1,187,760	2.966.546
South Kalimantan	392,750	–	570,802	46,393	1,009,945
Central Kalimantan	571,895	90,884	1,488,642	2,459,556	4,610,977
East Kalimantan	710,835	4,845	75,206	176,963	967,850
North Kalimantan	437,619	11,967	51,262	204,977	705,825
Total	3,045,938	467,813	2,671,743	4,075,649	10,261,143

The utilization of tidal swamps for rice farming can be done by opening and developing new rice fields. The Indonesian government has implemented a program to open new rice fields from 2015 to 2019. The total area of new open rice fields until 2019 is 222,442 ha [7]. Meanwhile, in West Kalimantan, new paddy fields have been created in several districts, and the area has reached 23,384 ha [8] (Table 3). However, the area planted with rice is still relatively small, i.e. around 30%.

**Table 3:** Distribution of newly-opened paddy fields in West Kalimantan.

Number	Regency	Area (ha)
1.	Sambas	1,490.00
2.	Kubu Raya	2,000.00
3.	Bengkayang	1,000.13
4.	Landak	5,222.07
5.	Sanggau	6,840.02
6.	Sekadau	852.53
7.	Melawi	465.57
8.	Sintang	2,315.00
9.	Kapuas Hulu	1,799.10
10.	Ketapang	1,400.00
	Total	23,384.42

Efforts to expand new rice fields for rice farming in the tidal swamps, especially in the acid sulphate soils, are still facing obstacles, such as low soil fertility level. Several studies have been conducted regarding low soil fertility in the newly-opened tidal rice fields. However, there is still lack of site-specific technology for rice farming management in the newly-opened tidal paddy fields based on land biophysical characteristics, socio-economic and institutional requirements that can support sustainable rice farming. Therefore, this study was aimed to perform a sustainable rice farming model in the newly-opened tidal rice fields.

## 2 Methods

The research was conducted in Sambas and Mempawah Regencies which have newly-opened paddy fields. Sambas Regency has 1,490 ha of newly-opened paddy fields in 2017.

This research used a survey method. Methods of data collection were structured interviews and focus group discussions. The data and information used in this study including primary and secondary data. Sampling method was purposive samples. Primary data included land biophysics and farmers’ socio-economic condition that were obtained by interviewing some respondents. The respondents consisted of individual farmers in the newly-opened paddy fields, farmer groups, combined farmer groups (“Gapoktan”), cooperatives, village-owned enterprises (“BUMDes”), agricultural extension officers, district agriculture service employees, employees of “Bank Rakyat Indonesia (BRI)”, employees of “Bank Indonesia (BI)”, and private companies.

The total number of farmers who were involved in this study were 60 farmers from Sambas Regency and 50 farmers from Mempawah Regency. Nevertheless, farmers that were chosen to be interviewed for data collection were 50 farmers. They were 25 farmers from Sambas District and 25 farmers from Mempawah District. Those 50 respondent farmers from the study area were purposively selected based on their active participation in rice cultivation on the newly-opened paddy fields.

Data related to the structure of community profiles and institutional profiles were obtained through in-depth interviews and field observations. The collection of data and information on institutional models was carried out through focus group discussions (FGD). Whereas secondary data such as planting area, harvest area, and new superior rice variety production were obtained from Agricultural Agencies in Sambas and Mempawah Regencies.

Data analysis on the socio-economic condition of the respondents used descriptive statistical analysis using SPSS version 20. The design of the newly-opened rice farming model was carried out using the FGD method, which involved all stakeholders in the newly-opened rice fields area.

## 3 Results and discussions

### 3.1 Requirements for rice farming in the newly-opened paddy fields

#### 3.1.1 Biophysical requirements for land and rice cultivation

The biophysical requirements of land include water availability, drainage, and soil chemical properties (cation exchange capacity, salinity, alkalinity, pyrite). In general, rice cultivation in the newly-opened tidal fields has some problems, such as low soil productivity due to toxic concentrations of Fe, Al, and Mn and nutrients deficiencies of P, K, Ca, Mg, and Zn [9].

Based on the results of past studies about the newly-opened rice fields in 2014 and 2015 growing seasons that was located on Sungai Nipah Village, Mempawah District, West Kalimantan and in 2018 growing season in Sungai Daun Village, Sambas District, West Kalimantan, we found that rice farming in the newly-opened rice fields can be carried out by managing water drainage, i.e. washing the land to reduce the solubility of toxic elements [9,10]. In addition, managing nutrients through balanced fertilization, applying amelioration, and cultivating rice with the appropriate techniques play significant role in rice farming management [9,10].

The need for waterlogging and drainage in the newly-opened rice fields is still not performed. Physicochemical processes in the newly-opened rice fields still occur, in addition to iron and manganese toxicity [11]. Specific cultivation technology such as soil amelioration based on the conditions and characteristics of the land should be applied to control the toxic Fe^2+^ [10]. In the conditions of continuous reduction or flooding, the newly-opened rice fields will increase the availability of iron oxide at certain concentrations which can be toxic to rice plants. The concentration of iron oxide after soaking the newly-opened rice fields for 3 to 4 weeks increases by about 600 ppm [12]. The critical concentration of iron oxide which can be toxic to rice plants is around 60–300 ppm [13]. The reaction in acidic soil with a pH of 5.92 can cause Fe in a reduced form [14]. The reduction of iron from Fe^3+^to Fe^2+^occurs in the immersion of newly-opened paddy soil, which can reach 1,121.14 ppm and is classified as very high [15]. High concentrations of iron in soil solutions can cause iron toxicity that can damage rice plants. The iron toxicity symptoms can be identified by the slow growth of rice plants and rust spots on old leaves. The critical limit for iron toxicity for rice plants in tidal areas is 260 ppm [16]. Therefore, implementing a good irrigation system such as intermittent irrigation and one-way flow irrigation can reduce iron toxicity in the newly-opened rice fields [10].

Another requirement in managing the newly-opened paddy fields is to apply the appropriate rice cultivation technique. Soil tillage in paddy fields accelerates the formation of a plow layer [17]. Giving lime to newly-opened rice fields can increase the pH of the soil and seeds of rice plants [18]. Balanced fertilization by analyzing the status of soil nutrients, especially P and K, can be a recommendation for site-specific fertilization [19]. The application of a combination of inorganic fertilizers, biochar, rice straw compost, manure, and Zn is recommended in paddy fields to increase plant growth [20].

The results of past studies in Sungai Nipah Village, Mempawah Regency, West Kalimantan, Indonesia showed that management of the newly-opened tidal paddy fields for rice farming was very specific, in particular, different water management in the dry season of 2014 and rainy season of 2015 (Table 4) [10]. Besides, rice yields were significantly affected by rice varieties, as demonstrated in the past study of 2018 in Sungai Daun Village, Sambas Regency, West Kalimantan, Indonesia (Table 5) [9].

**Table 4:** Effect of organic fertilizer on vegetative growth and rice yield at Sungai Nipah village, Mempawah Regency, West Kalimantan, Indonesia, 2014 and 2015 [10].

Treatment	Plant height (cm)	Panicle lenght(cm)	Panicles (number/clump)	Grains (number/panicle)	Unfilled grains (number/panicle)	Filled grains number/panicle)	1000-filled grains weight (g)	Yield (14% moisture content) (t.ha^−1^)
T0 = without ameliorant (control)	122,34 a^a^	25,82 a	6,64 a	95,62 a	74,22 a	21,40 a	18,24 a	2.27 a
T1 = chicken manure	123,12 a	38,68 b	12,85 b	145,35 b	118,25 b	27,10 b	20,34 a	4.32 b
T2 = dolomite	122,64 a	35,54 b	12,30 b	134,69 b	112,34 b	22,35 b	20,26 a	4.30 b
T3 = biochar	125,32 a	38,32 b	18,86 c	180,40c	144,80 c	35,6 b	21,84 a	5.76 c

a The numbers in the same column followed by the same letter are not significantly different at the 5% level based on the DMRT test.

**Table 5:** Rice varieties and productivity in the newly-opened paddy fields in Sungai Daun Village, Sambas Regency, West Kalimantan, Indonesia, in 2018 [9].

Variety	Productivity of dry grain (t.ha^−1^)
V1 = Sokan (control or farmer)	2,10 a^a^
V2 = Kristal	4,22 b
V3 = Inpari 34	5,72 c
V4 = Margasari	5,42 c
V5 = Inpari 35	2,62 a

a The numbers in the same column followed by the same letter are not significantly different at the 5% level based on the DMRT test.

The results of the study about organic fertilizer affected vegetative growth and rice yield in the newly-opened paddy fields in tidal swampy land (Table 4), it showed that some ameliorant input was needed for soil improvement, so that the rice crops could have better agronomic performance during the vegetative and generative periods, and then resulted in greater production compared to no ameliorant [10]. Variety of soil ameliorants can be adjusted based on availability in the surrounding farms such as manure, in-situ plant residue, or biochar.

Several rice varieties affected rice productivity (Table 5). The Sokan variety, which was a local rice commonly grown by local farmers and was the control treatment in this study, had lower productivity compared to superior varieties, except for Inpari 35. This condition indicated that high yielding varieties were suitable for planting in the newly-opened rice fields, to obtain high productivity.

Specific cultivation technology was required for growing rice during the dry season. It was necessary to have intermittent irrigation, i.e. drainage and flooding for one week each; while in the rainy season, it needed 1-week flooding and 2 weeks of drying. Amelioration and intermittent irrigation for one week each in the dry season increased rice dry grains yield by 237.14% compared to control [10]. In the rainy season, the increasing grain yield reached 271.90% with the technological packages consisting of 2 weeks of drying and one week of flooding, followed by ameliorant, organic, and inorganic fertilizer applications [10].

Some studies regarding increased rice productivity in the newly-opened swampy land reported that the application of urea, SP-36, and KCl at 250, 100, and 100 kg ha^−1^, respectively, 2 t ha^−1^ of dolomite, and 2 t ha^−1^of compost increased rice grain yield by 1.78 t ha^−1^ in the newly-opened paddy fields in Bulungan District [21]. The arrangement of diverting water layers in the newly-opened paddy fields significantly increased rice grain yield from 3.37 to 4.47 t ha^−1^ with the enhancement about 0.47–1.10 t ha^−1^. Furthermore, [22] added that integrated crop management of Inpara 3 and Inpari 30 rice varieties planted with a 4:1 path row planting system (locally known as “jajar legowo”), incorporated with 1 t ha^−1^ organic matter and balanced fertilization based on soil test kit were able to increase rice yield in the paddy fields [23].

In summary, recommendations for rice cultivation in the newly-opened paddy field are using adaptive superior rice varieties, controlling pests and diseases, and properly harvesting rice in order to reduce yield loss in paddy fields.

#### 3.1.2 Socio-economic and institutional requirements

Socio-economic and institutional requirements for rice farming in the newly-opened paddy fields play an important role in sustainable rice farming. Based on the results of this study from the interview and FGD, we identified that these requirements include the availability of human resources (farmers), agricultural machinery, capital, and farmer economic institutions.

Human resources are the existence of farmers and farmer groups who manage the newly-opened paddy fields. Human resource management is one of the most complex challenges in agro-food enterprises and plays a significant role in the agriculture and food processing sector. It is impacted more by social than by economic drivers [24]. As also stated by Ref. [25]that agribusiness organizations are successful because of their human resources, and human resources management is a strategy that aids businesses in achieving sustained competitive advantage.

In managing rice plants, farmers are expected to establish and cooperate into a farmer group. A farmer group is an organization that accommodates farmers in managing their farms with similar needs and goals. Effective management and development of farming systems can be carried out through a land group approach to increase the capacity of their farms [26].

Farmers in groups must be open to receiving new ideas such as rice cultivation technology in the newly-opened paddy fields and must be able to collaborate with stakeholders in implementing technology guidance and field schools. Farmer group leaders play a significant role in the dynamics and progress of farmer groups, as stated by Ref. [27]. Increasing the educational capacity of farmer group leaders is important to enhance farmer group dynamics.

Farmers' capital in rice farming is often the main obstacle, especially in the provision of agricultural production facilities such as seeds, ameliorants, fertilizers, pesticides, agricultural tools, and machinery. The research by Ref. [28] revealed that farmers in several African countries experienced difficulties in managing their farms, especially in increasing farming capacity, due to lack of capital and farm inefficiency, so in the end, farmers have some constraints in managing their farm households.

Newly-opened paddy fields usually have low productivity; thus, it needs a lot of input to increase soil fertility. Based on research in several locations of the newly-opened paddy fields in West Kalimantan, farmers are not equipped with sufficient knowledge to manage their rice fields so the yield is not optimal or considered very low (below 2 t ha^−1^) [10]. Therefore, farmers in the following growing season did not plant rice, and the newly-opened paddy fields became dormant land (fallow) for a long time.

The Indonesian government has provided agricultural facilities and infrastructure in the newly-opened rice fields, agricultural equipment and machineries such as tractors/hand tractors, and fertilizer subsidies to farmer groups; however, based on the observation in the study area, it is still insufficient to increase rice productivity of the newly-opened paddy fields in Mempawah and Sambas Regencies, West Kalimantan. Farmers still need intensive technological guidance from researchers and agricultural extension workers as well as technicians on proper rice cultivation techniques in the newly-opened paddy fields. In addition [29], asserted that government programs requiring bottom-up and not top-down. An approach that does not come from the farmers themselves will lead to false participation of farmers in the programs [29].

The involvement of the private sector and banks is also very important in helping farmers or farmer groups to cultivate rice in the newly-opened paddy fields in Mempawah and Sambas Regencies, West Kalimantan, Indonesia, such as ensuring good rice yields and unhulled rice prices and providing capital for the procurement of agricultural production facilities through a farming credit scheme without assurance and low interest such as Agricultural People's Business Credit (“KUR”) provided by the Indonesian government. The government's role in supporting the smooth running of financial institutions and marketing of agricultural commodities is very important to make it easier for farmers to get loans from banks and market their crops [30].

The results of this study in West Kalimantan indicated that farmers' level institutions are the main key in rice farming in the newly-opened rice fields, so that it can be sustainable. Furthermore, the availability of farmer groups, farmer economic institutions, cooperatives, and village-owned enterprises needs to be improved, formed, initiated, and managed properly. The research by Ref. [31] in India, revealed that farmers as members of farmer groups will find it easier to market their crops and get other benefits compared to farmers who are not members of farmer groups. In relation to that result, in the swampland area in South Kalimantan, farmers satisfaction value with the performance of farmer groups was 67.21%. However, they were ought to make some priority in organizing internal management in the farmer groups, so that they can achieve successful work in agricultural program and enhance farmers’ welfare. Farmers had to improve some efforts in Statutes/Articles of Association, vision, and mission, organizational structure, work plan, administration, financial records, training, superior seed, integrated pest control, granting credit and the latest information [32]. In addition [33], discovered that organized farmers observed higher benefits than unorganised smallholders. Moreover, farmers who joined in a farmer group and a cooperative obtained better benefits than farmers who only involved in farmer groups [33].

### 3.2 Development of rice farming model in the newly-opened tidal rice fields in Indonesia

**Fig. 2:**
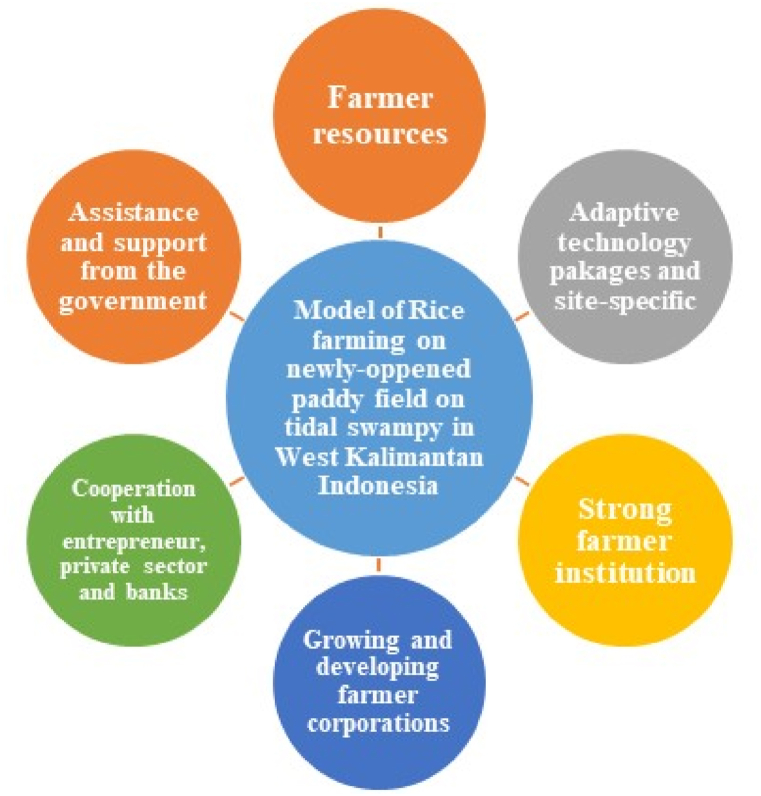
Suggested rice farming models in the newly-opened tidal rice fields.

Development of a rice farming model in the newly-opened tidal rice fields, such as the case in West Kalimantan, is not established partially which only involves land biophysical factors, but it is a comprehensive model involving all factors that can affect a successful and sustainable rice farming. This model consists of a combination of farmer groups, agricultural researchers and extension agents, government agencies, the private sector, and banks (Fig. 2).

The development of this farming system model is the result of several research and assessments that had been conducted by the interdisciplinary research team for 3 years from 2017 to 2019 through the Sustainable Management of Agricultural Research and Technology Dissemination (SMARTD) project arranged by the Indonesian Agency for Agricultural Research and Development (IAARD) of the Ministry of Agriculture of the Republic of Indonesia. This research was started with the adaptation of new superior rice varieties, application of local-specific technology packages, assessment of farming systems, and studies of farmer economic institutions. This model is packaged in various subsystem forms ranging from rice cultivation, banking-supported capital, pre-production, production, harvest, and post-harvest subsystems, in addition to product distribution and marketing. The development model of the newly-opened rice farming in the tidal swamp for the case in West Kalimantan is as follows.

#### 3.2.1 Human resources (farmers)

At the location of the newly-opened tidal paddy fields in Sungai Daun Village, Selakau District, Sambas Regency, West Kalimantan Province, Indonesia, some farmers manage their farms in groups who are members of two farmer groups under the names “Berkat Tani” and “Tani Jaya.” Whereas, the two farmer groups who were involved in this study from Sungai Nipah Village, Mempawah Regency were farmer groups of “Karya Tani” and “Usaha Tani.”

Each farmer group in Sambas Regency has 30 members, while in Mempawah Regency each farmer group has 25 members. All farmers worked together and committed to implementing rice farming technology provided by researchers and agricultural extension agents from the Assessment Institute for Agricultural Technology (AIAT) of West Kalimantan and the Agricultural Service and other related agency technicians.

#### 3.2.2 Adaptive local specific technology package

The applied technology package has been tested through a process of adaptation research, and research on local-specific technology components in the newly-opened rice fields area (village) of 50 ha.

The adaptive superior rice varieties of Inpari 34 and Margasari with a one-way flow micro water management, site-specific balanced fertilization from the results of soil nutrient status analysis, and the provision of organic matter from rice straw compost of 5 t ha^−1^, resulting in the average rice productivity of 5.7 t ha^−1^ (Inpari 34) and 5.54 t ha^−1^ (Margasari). Meanwhile, the rice productivity of locals was at an average of 2.1 t ha^−1^. It shows that there was an increase in rice productivity with the application of the rice cultivation technology package in the newly-opened tidal rice fields of 3.5 t ha^−1^ and thus, farmers' income increased by 10.6 million rupiahs.

#### 3.2.3 Requirement of strong farmer organizations for sustainable rice farming

Farmer institutions such as farmer groups or farmer group associations are willing to accept technological information input and adopt useful technology, especially rice farming in the newly-opened rice fields. Regular technical guidance and field schools are continuously provided to farmer groups. Good cooperation continues between members of the farmer groups.

In this regard, a Farmers' Economic Institute (“KEP”) was established, in the case of West Kalimantan, a Village-Owned Enterprise (“BUMDes”) was established under the name of “Usaha Bersama,” to strengthen and empower the rural economic institutions.

Farmers' economic institutions need to commit to supporting rice farming, such as capitalization and product marketing. In this case, the “BUMDes Usaha Bersama” cooperates with farmer groups, provides agricultural production facilities (seeds, fertilizers, pesticides, and ameliorant materials), and buys rice yields from farmer groups based on a mutual agreement.

“BUMDes Usaha Bersama" collects unhulled rice from farmer groups and then mills and sells the rice to the market and labels it as quality rice, which is processed in collaboration with the Rice Milling Unit (RMU) belongs to a village farmers' group.

#### 3.2.4 Growing and developing farmer corporations

Growing farmer corporations starts from the process of introducing technology packages and models in definite locations, determining locations, consolidating cross-stakeholder, farmer and farm, designing farmer corporate and business models, determining farmer economic institutions, management, and legal status, processing infrastructure readiness and human resources.

Developing farmer corporations, among others; processing technical guidance, training activities, business network, and loan application to formal financial institutions (banks).

#### 3.2.5 Assistance from government and related agencies

Sustainable technology assistance and guidance from researchers, agricultural extension workers, and pest and plant disease control officers were observed as a great support.

Additionally, support from related agencies in providing facilities and infrastructure, such as farming roads, irrigation, drainage channels, agricultural equipment and machinery, and marketing (Farmers Market/Farmers Shop) was highly beneficial as a continuous support.

Assistance for farmer groups through a combination of farmer groups has been provided by the local government, i.e. tractors, threshers, repair of waterways, and farm roads [8].

#### 3.2.6 Cooperation with entrepreneurs, the private sector, and banks

It is suggested to provide cheap farm credit assistance without assurance from the government banks such as “BRI” Bank or “BNI” Bank in Sambas District, among others, the Agricultural Credit (“KUR”) Program with an interest rate of 6% per year, a maximum credit limit of IDR 50 million with flexible loan terms of 12, 18, and 24 months.

KUR or People's Business Credit is a government program regarding the provision of capital with a credit system that aims to help Micro, Small and Medium Enterprises (MSMEs) or farmers overcome capital difficulties. Each type of business has different guarantee from the government, namely 80% of the credit ceiling for businesses in the fields of forestry, agriculture, fisheries, and small industries [34].

The “KUR” Program was implemented by the Indonesian government since August 2015 using the interest/margin subsidy scheme. Interest subsidy is the portion of interest that is the burden of the government in the amount of the difference between the interest rate received by the credit/financing dealer and the interest rate charged to the debtor. Meanwhile, margin subsidy is the part of the margin that is the burden on the government in the amount of the difference between the margin received by the credit/financing distributor and the margin charged to the debtor in the Sharia financing scheme. The provision of interest/margin subsidies causes the interest rate for KUR loans/financing to be very low compared to commercial banking loans. The interest rate continued to decline from 2008 by 24% and then continued to decline until 2020 at the level of 6% [35].

Minister of Agriculture developed Regulation No. 40 in 2015 about agricultural insurance facilitation [36]. Agricultural insurance was aimed to protect farmers from crop failure due to farming risks such as flooding, drought, and pest outbreak or poor harvest years [37]. PT. Jasindo Insurance would be helpful as an agricultural insurance company [38]. In this regard, PT. Jasindo can bear the losses of farmers due to crop failure. In rice farming insurance, the amount of the claim fee for damage to rice farming is 75%, which is IDR 6,000,000/ha/season. The premium paid is IDR 180,000/ha/season. The amount of premium support from the government is IDR 144,000/ha/season and the rest are self-subsistent farmers of IDR 36,000/ha/season [38],[36]. Recognition of government subsidies and farmer insurance benefits is beneficial, enhances agricultural prospects and helps farmers maintain their livelihoods [39,40,41].

Minister of Agriculture developed Regulation No. 40 in 2015 about agricultural insurance facilitation [36]. Agricultural insurance was aimed to protect farmers from crop failure due to farming risks such as flooding, drought, and pest outbreak or poor harvest years [37]. PT. Jasindo Insurance would be helpful as an agricultural insurance company [38]. In this regard, PT. Jasindo can bear the losses of farmers due to crop failure. In rice farming insurance, the amount of the claim fee for damage to rice farming is 75%, which is IDR 6,000,000/ha/season. The premium paid is IDR 180,000/ha/season. The amount of premium support from the government is IDR 144,000/ha/season and the rest are self-subsistent farmers of IDR 36,000/ha/season [38],[36]. Recognition of government subsidies and farmer insurance benefits is beneficial, enhances agricultural prospects and helps farmers maintain their livelihoods [39,40,41].

The cooperation between farmer groups and PT. Sandong is possible in terms of providing agricultural production facilities and is suggested marketing the rice yield.

**Fig. 3:**
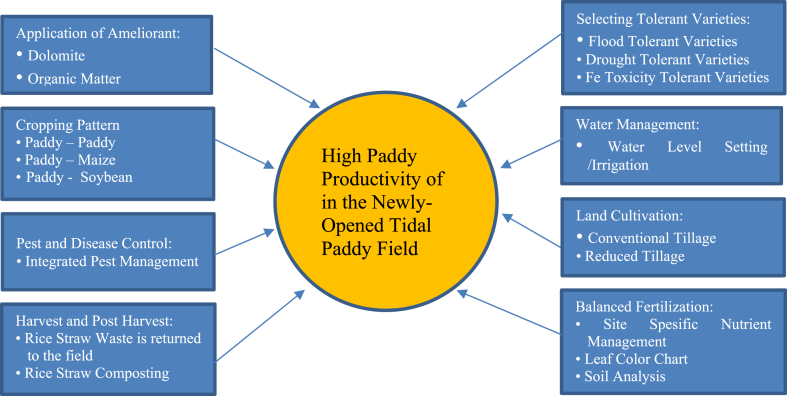
Model of high paddy productivity in the newly-opened tidal paddy field.

Overall, high paddy productivity can be achieved through implementation of the model of the newly-opened tidal paddy field as described in Fig. 3.

Limitations that need to be improved in the newly-opened tidal paddy field model for rice farming are as follows:

1. A lot of research has been conducted on increasing rice productivity in the newly-opened tidal paddy fields; nonetheless, the approach to resolving the problems in the field has not been integrated across sectors. There are still some drawbacks in the field, such as irrigation system damage, lack of adaptive rice varieties for acidic soils, Fe toxicity, and long-term drought, as well as external factors such as the socio-economic conditions of farmers.
2. Vulnerable and ineffective farmer organization. Farmer organization should be properly managed to play a functional role in the agricultural system and to facilitate farmers in agriculture financially.
3. Limited role of extension agents that resulted in limited access on agricultural innovation technology for farmers in the area of newly-opened tidal paddy fields. Established and effective farmers' group can be an intermediate agents to overcome this drawback. Well-organized farmer groups are well-acquainted with their members' needs. They would have direct access to the entire members, and can act as channels for horizontal learning and information sharing between different members' groups, especially related to innovative technology in agriculture and farmers' knowledge gaining experiences.

## 4 Conclusion

Management of the newly-opened tidal rice fields requires good cooperation between farmer groups, farmer economic institutions, related agencies, local government, the private sector, and banking, in addition to technological guidance from researchers and agricultural extension agents.

The rice farming model in the newly-opened tidal lowland rice field in the West Kalimantan case is expected to be replicated in other areas in Indonesia, in which 222,442 ha area of the newly-opened rice fields have a potential yield of 1.2 million tons of rice per year with the assumed average yield of 5 t ha^−1^. This potential yield can support the national food self-sufficiency program by the application of the suggested model.

## Author contribution statement

Muhammad Hatta: Conceived and designed the experiments; Performed the experiments; Analyzed and interpreted the data; Wrote the paper.

Sulakhudin: Conceived and designed the experiments; Wrote the paper.

Rusli Burhansya; Gontom C. Kifli: Analyzed and interpreted the data.

Dina O. Dewi; Putri Tria Santari: Performed the experiments.

Juliana C. Kilmanun; Dadan Permana; Khojin Supriadi; Hozin Azis: Contributed reagents, materials, analysis tools or data.

Riki Warman: Performed the experiments; Wrote the paper.

Dwi P. Widiastuti: Conceived and designed the experiments; Analyzed and interpreted the data; Wrote the paper.

## Funding statement

We would like to acknowledge all colleagues for extensive support in this research. Finally yet importantly, we would like to express our gratitude to Indonesian Agency for Agricultural Research and Development for financial support.

## Data availability statement

Data included in article/supplementary material/referenced in article.

## Declaration of interest’s statement

The authors declare no conflict of interest.

## References

[1] Udmale P., Pal I., Szabo S., Pramanik M., Large A. (2020). Global food security in the context of COVID-19: a scenario-based exploratory analysis. Prog. Disas. Sci..

[2] Priyadarshini P., Abhilash P.C. (2021). Agri-Food systems in India: concerns and policy recommendations for building resilience in post COVID-19 pandemic times. Global Food Secur..

[3] FAO (2023). https://www.fao.org/faostat/en/#data/QCL/visualize.

[4] Statistics Indonesia (2020).

[5] Statistics Indonesia (2021).

[6] Indonesian Center for Agricultural Land Resources Research (2020).

[7] Reza (2020). https://www.liputan6.com/bisnis/read/4242930/dalam-periode-2015-2019-kementan-telah-cetak-sawah-baru-sebanyak-224977.

[8] Department of Food Crops and Horticulture West Kalimantan Province (2017).

[9] Hatta M., Sulakhudin (2019). Amelioration effect of rice productivity on newly-opened paddy field in West Kalimantan. IOP Conf. Ser. Earth Environ. Sci..

[10] Sulakhudin, Hatta M. (2018). Increasing productivity of newly-opened paddy field in tidal swampy areas using a local specific technology. Indones. J. Agric. Sci..

[11] Widowati L.R., Sukristyonubowo (2019). Dynamics of pH, ferrum and mangan, and phosphorus on newly-opened paddy soil having high soil organic matter on rice growth. J. Trop. Soils.

[12] Utama M.Z.H., Wahidi I., Sunadi (2013). Response of some rice cultivars in new opening paddy fields with high Fe2+ using multi-packet technology. J. Trop. Soils.

[13] Mahender A., Mallikarjuna Swamy B.P., Anandan A., Ali J. (2019). Tolerance of iron deficient and toxic soil conditions in rice. Plants.

[14] Ritvo G., Avnimelech Y., Kochba M. (2003). Empirical relationship between conventionally determined pH and in situ values in waterlogged soils. Aquacult. Eng..

[15] Widowati L.R., Sleutel S., Setyorini D., De Neve S. (2012). Nitrogen mineralization from amended and unamended intensively managed tropical Andisols and Inceptisols. Soil Res..

[16] Rumanti I.A., Hairmansis A., Nugraha Y., Nafisah Susanto U., Wardana P., Subandiono R.E. (2018). Development of tolerant rice varieties for stress-prone ecosystems in the coastal deltas of Indonesia. Field Crop. Res..

[17] Jeřábek J., Zumr D., Dostál T. (2017). Identifying the plough pan position on cultivated soils by measurements of electrical resistivity and penetration resistance. Soil Tillage Res..

[18] Santri J.A., Maas A., Utami S.N.H., Yusuf W.A. (2019). Application of lime and compost on the newly established field with acid sulfate soil type in the Belandean Experimental Field, South Kalimantan for agricultural cultivation. IOP Conf. Ser. Earth Environ. Sci..

[19] Hatta M. (2020). A site-specific fertilizer recommendation based on the phosphorus and potassium status in Mempawah district, West Kalimantan. J. Trop. Soils.

[20] Abbhishek K., Chander G., Dixit S., Kuttippurath J., Singh A., Das D. (2021). Legume biochar fertilizer can be an efficient alternative to compost in integrated nutrient management of paddy (*Oryza Sativa* L.). J. Soil Sci. Plant Nutr..

[21] Sukristiyonubowo, Nugroho K., Vadari T. (2012). Nutrient removal by rice cultivated in newly-opened wetland rice in Bulungan District, East Kalimantan. J. Trop. Soils.

[22] Mildaerizanti, Handoko (2016). http://pur-plso.unsri.ac.id/userfiles/43_HAL%20543-547_Mildaerizanti.pdf.

[23] Paiman A., Ansar M., Effendy I., Sumbodo B.T. (2020). Rice cultivation of superior variety in swamps to increase food security in Indonesia. Rev. Agri. Sci..

[24] Ratkovic T. (2015). HRM in foreign-owned agricultural and food processing companies in Serbia. Econom. Agri..

[25] Konja V., Uzelac O. (2015).

[26] Martin G. (2015). A Conceptual framework to support adaptation of farming systems – development and application with forage rummy. Agric. Syst..

[27] Ram D., Ganpat W., Narine L.K. (2017). Management performance of farmers groups and its impact on membership: a prerequisite for group sustainability in Trinidad. J. Agric. Ext. Rural Dev..

[28] Poole N.D., Chitundu M., Msoni R. (2013). Commercialisation: a meta-approach for agricultural development among smallholder farmers in Africa?. Food Pol..

[29] Nourani V., Maertens A., Michelson H. (2021). Public good provision and democracy: evidence from an experiment with farmer groups in Malawi. World Dev..

[30] Zhang D. (2020). The innovation research of contract farming financing mode under the block chain technology. J. Clean. Prod..

[31] Agarwal B. (2018). Can group farms outperform individual family farms? Empirical insights from India. World Dev..

[32] Marhamah B.T., Masyhuri, Waluyati L.R. (2020). Farmer satisfaction through farmer group performance: a study in swampland, South Kalimantan. J. Appl. Manag..

[33] Ibnu M., Offermans A., Glasbergen P. (2018). Certification and farmer organisation: Indonesian smallholder perceptions of benefits. Appl. Artif. Intell..

[34] National Standardization Agency of Indonesia (2022). https://www.bsn.or.id/tabel-kur-bri/.

[35] Coordinating Ministry for Economic Affairs (2023). https://kur.ekon.go.id/gambaran-umum.

[36] Silaban B., Burhanuddin, Harmini (2022). The impact of rice farm insurance on the income of farmers in Indonesia. J. Manaj. Agrib..

[37] Pasaribu S.M. (2010). Developing rice farm insurance in Indonesia. Agri. Agric. Sci. Proced..

[38] Ministry of Agriculture (2018).

[39] Islam M.D., Rahman A., Sarker M.S.R., Luo J., Liang H. (2021). Factors affecting farmers' willingness to adopt crop insurance to manage disaster risk: evidence from Bangladesh. Int. Food Agribus. Manag. Rev..

[40] Jin J., Wang W., Wang X. (2016). Farmers' risk preferences and agricultural weather index insurance uptake in rural China. Int. J. Disas. Risk Sci..

[41] Nain M.S., Singh R., Mishra J.R. (2017). A study of farmers' awareness on agricultural insurance schemes in Southern Haryana. Ind. J. Extens. Educ..

